# Downregulation of sperm-associated antigen 5 inhibits melanoma progression by regulating forkhead box protein M1/A disintegrin and metalloproteinase 17/NOTCH1 signaling

**DOI:** 10.1080/21655979.2022.2031670

**Published:** 2022-02-09

**Authors:** Lin Dang, Cuiping Shi, Qianqian Zhang, Peiyu Liao, Yan Wang

**Affiliations:** a Department of Dermatovenology, Shenzhen People’s Hospital, The Second Clinical Medical College of Jinan University, The First Affiliated Hospital of Southern University of Science and Technology, Shenzhen, China; b Department of Pathology, Shenzhen People’s Hospital, The Second Clinical Medical College of Jinan University, The First Affiliated Hospital of Southern University of Science and Technology, Shenzhen, China

**Keywords:** Melanoma, cell growth, migration, Sperm-associated antigen 5, forkhead box protein M1

## Abstract

Sperm-associated antigen 5 (SPAG5) has been identified as a driver in several type of cancers. In this study, we aimed to reveal the role of SPAG5 in melanoma and clarify whether FOXM1 (forkhead box protein M1) /ADAM17 (A disintegrin and metalloproteinase 17) /NOTCH1 signaling was involved. The expression of SPAG5 in malignant melanoma (MM) tissues and matched normal tissues was detected using qRT-PCR, immunohistochemistry and Western blotting. Cell viability was tested using CCK-8 (Cell Count Kit-8), colony formation and EdU staining. Cell migration and epithelial to mesenchymal transition (EMT) were measured using transwell chambers and immunofluorescent staining. Cell cycle distribution and tumorigenesis were assessed by flow cytometry and in vivo tumor-bearing experiments, respectively. The results demonstrated that the expression of SPAG5 was increased in MM tissues and cells. Downregulation of SPAG5 inhibited cell viability, migration, invasion and EMT, and induced a G1-phase arrest. In addition, downregulation of SPAG5 decreased the expression of FOXM1, thereafter inhibiting the expression of ADAM17, NOTCH1 and HES1. Furthermore, deletion of SPAG5 expression decreased the tumorigenesis of MM A375 cells. In conclusion, this study demonstrated that SPAG5 was overexpressed in MM. Downregulation of SPAG5 repressed MM cell growth and EMT, which might be induced by inactivation of the FOXM1/ADAM17/NOTCH1 signaling.

## Graphical abstract

**Figure:**
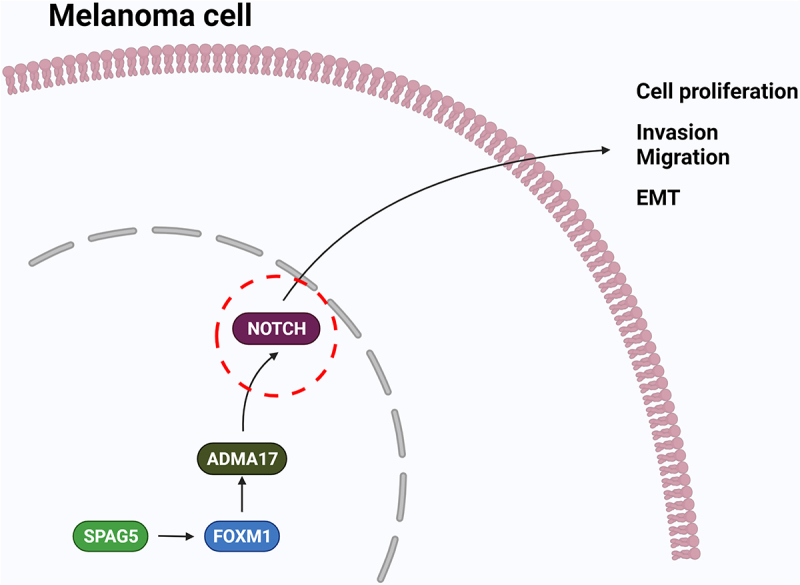

## Introduction

Malignant melanoma (MM) is a highly aggressive tumor and has the highest fatality rate among skin cancers due to the high metastatic rate [1,2]. Currently, the 5-year survival rate for melanoma patients at early stage is 92%, which drops to only 15% when it metastasis [3]. Epithelial-mesenchymal transition (EMT) is a common phenomenon occurring in cancer cells, including MM [4], which can enhance cell migratory capacity, increase invasiveness, and suppress apoptosis [5,6], highlighting it as a potential therapeutic target in melanoma.

Sperm-associated antigen 5 (SPAG5), also called as DEEPEST, MAP126, hMAP126, is a mitotic spindle-associated protein which is responsible for centrosome integrity and the maintenance of sister chromatid cohesion and during mitosis [7]. SPAG5 deficiency in rat hypogonadic testes can lead to cell death with disorganization of the spindle apparatus [8]. Also, deletion of SPAG5 facilitates the formation of disordered spindles and triggers growth arrest [9]. Recently, growing evidence has demonstrated that SPAG5 plays a role in cancers. For example, SPAG5 was significantly overexpressed in triple-negative breast cancer (TNBC) tissues, and overexpression of SPAG5 promoted tumor growth *in vitr*o and *in vivo* [10]. Also, SPAG5 was reported to overexpress in cervical cancer [11], prostate cancer [12], gastric cancer [13] and lung cancer [14], and contributed to carcinogenesis. However, the role of SPAG5 in MM remains unknown.

The forkhead box protein M1 (FOXM1) transcription factor has been reported to express at higher levels in metastatic melanoma [15,16], and is closely implicated in the regulation of many biological processes, such as cell proliferation, apoptosis and cell cycle [17]. FOXM1 is positively regulated by SPAG5 [18], and also promotes the expression of ADAM17 (A disintegrin and metalloproteinase 17), which belongs to the membrane-bound enzyme family responsible for cleaving cell surface proteins, such as cytokines, cytokinereceptors and adhesion proteins [19,20]. Moreover, ADAM17 serves as a positive regulator of NOTCH signaling and is involved in many types of carcinogenesis [21–23]. These findings suggest that SPAG5 might play a role in regulating ADAM17/NOTCH signaling through FOXM1. Furthermore, studies have demonstrated that ADAM17 was significantly unregulated in melanomas [24,25], and downregulation of ADAM17 induced by rosmarinic acid resulted in inhibitions in cell growth and migration, and increases in cell apoptosis and cisplatin sensitivity in melanoma cells [25], indicating that ADAM17 serves as an oncogene in MM. Thus, we conjectured that SPAG5 may be involved in MM progression through regulating FOXM1/ADAM17/NOTCH signaling. However, currently, no reports have verified this hypothesis.

In this study, we assumed that SPAG5 was deregulated in MM and implicated in MM progression through regulating the FOXM1/ADAM17/Notch signaling. To this end, the present study was carried out with two main goals: one is to explore SPAG5 expression profiles in melanoma, and another is to reveal whether SPAG5 involves in melanoma progression through regulating the ADAM17/Notch signaling via FOXM1.

## Materials and methods

### Clinical tissue samples and cell lines

Sixty MM tissues and the adjacent normal tissues were randomly obtained from patients with primary MM. All patients received surgical resection prior to any chemotherapy or radiotherapy, and tumor tissues and surrounding healthy tissue (within 1 cm around tumor) were collected during the operation. Inclusion criteria: (1) Patients with a new histological diagnosis of primary MM; (2) Patients received surgical resection prior to any chemotherapy or radiotherapy; (3) Fresh tumor tissues and the adjacent tissues that were preserved in liquid nitrogen; (4) Medical and follow-up records are complete and available; (5) Patients without other malignancies, serious illness or mental health problems. Exclusion criteria: (1) Patients received treatment prior to surgery, including chemotherapy and radiation therapy and other antitumor treatments; (2) Patients suffer from other malignant tumors, serious diseases or mental health problems. The age of the patients was between 35–64 years, with an average age of (56.8 ± 9.5) years. The clinical information of the 60 MM patients were shown in (Table 1). The written informed consents were obtained from patients or their parents. We got approval for this study from the Ethics Committee of Shenzhen People’s Hospital before this study.

**Table 1.** Patient characteristics for the cutaneous melanoma

Feature	Number of patient
Median age at diagnosis,years (range)	56.8
Gender	
Male	28
Female	32
Tumor thickness	
<1 cm	34
≥1 cm	26
TNM stage	
I–II	38
III–IV	22
Primary tumor histologic subtype	11 15 24 10 0
Superficia	
Nodular	
Acral Desmoplastic Other	
Metastasis	
Negative	36
Positive	24

### Immunohistochemistry

Immunohistochemistry (IHC) was performed to assess protein expression in cancer tissues according to previous report [26]. In brief, the formalin-fixed, paraffin-embedded tissues were cut into 4-μm sections, which were de-waxed, dehydrated, and retrieved by antigen-retrieval liquid. Then, the sections were blocked with 5% goat serum for 1 hour at room temperature, followed by incubation with indicated primary antibodies at 4°C overnight and second antibodies at 37°C for 30 min. The primary antibodies included anti-SPAG5 antibody (1:100 dilution, cat no. 14,726-1-AP, Proteintech, USA), anti-FOXM1 antibody (1:250 dilution, cat no. ab207298, Abcam, Cambridge, MA, USA), anti-ADAM17 antibody (1:100 dilution; cat no. ab39163, Abcam), ant-NOTCH1 antibody (1:150 dilution, cat no. ab52627, Abcam), anti-E-cadherin antibody (1:200 dilution; cat no. ab231303, Abcam) and anti-N-cadherin antibody (1:150 dilution; cat no. ab76011, Abcam). After that, the sections were reacted with diaminobezidin (DAB) for several seconds at room temperature and hematoxylin (Solarbio, Beijing, China) for 1 min. The staining was observed by using a microscope.

### Cell culture

Normal human dermal fibroblasts (NHDF, cat no. PCS-201-012) and four MM cell lines (A375, A2058) were purchased from Chinese Academy of Sciences Cell Bank (Shanghai, China). NHDF were cultured in Fibroblast Growth Kit–Low Serum (ATCC® PCS-201-041), supplemented with rhFGF (5 ng/mL), L-glutamine (7.5 mM), ascorbic acid (50 µg/mL), hydrocortisone hemisuccinate (1 µg/mL) and rhInsulin (5 µg/mL). A375 and A2058 cells were maintained in Dulbecco’s Modified Eagle’s Medium, and WM115 and MV3 cells were purchased from BeNa Culture Collection (Beijing, China) and grown in RPMI-1640 medium. All cell culture medium of MM cells were added 10% FBS (Fetal Bovine Serum) and 1% (v/v) penicillin/streptomycin. Cells were kept at 37°C in an atmosphere containing 5% CO_2_. Cell culture medium and FBS were obtained from Thermo Fisher Scientific (USA).

### Alternation of gene expression

To upregulate FOXM1 and ADAM17 expression, MM cells were transiently transfected with the overexpression plasmids of FOXM1 (cat no. RC202246, Origene, Beijing, China) and ADAM17 (cat no. SC316426, Origene) with the help of Lipofectamin 2000 (Invitrogen, USA) according to the manufacturer’s instructions.

The shRNAs used to downregulate SPAG5 (sh-SPAG5) in MM cells were synthesized by GenePharma (Shanghai, China), and were introduced into cells with 6 μg/ml of polybrene. The infected cells were kept in culture medium with 7 μg/ml of puromycin for 14 days to establish stable cell lines used in clone formation assay and animal experiments.

### Quantitative reverse transcription-PCR (qRT-PCR)

Total RNA extraction from tissues and cells was carried out by using TRIzol reagent (Invitrogen, USA) [27], and then subjected to cDNA synthesis with the help of PrimeScript RT Master Mix kit (RR036A; Takara). Next, the cDNA was applied to PCRs detection with 2× SYBR Green PCR Mastermix (Solarbio, Beijing, China) in e 7500 Real-Time PCR System (Applied Biosystems, USA). Primers were listed in (Table 2).

**Table 2.** Primer sequences

Gene	Sense (5’-3’)	Antisense (5’-3’)
SPAG5	CTGGTAGGGCTTCATGCCAA	TGCTGGCTCTTGACTGTGAG
β-actin	CTTCGCGGGCGACGAT	CCACATAGGAATCCTTCTGACC

### Western blotting

Protein expression was tested using Western blotting assay [26]. Total proteins from tissues and cells were isolated with the help of lysis buffer (Solarbio, Beijing, China) with 1% protease inhibitor (Solarbio). After centrifugation at 4°C for 30 min, Bicinchoninic acid Protein Assay kits (Thermo Fisher Scientific) were applied to examine protein concentrations in the light of specifications. Then, protein samples were loaded to 10% SDS-polyacrylamide gel and submitted to electrophoresis and transferred onto polyvinylidene difluoride membranes (PVDF; Millipore, Billerica, MA, USA). Next, the membranes were probed with primary antibodies overnight at 4°C after blocked in 5% nonfat milk for 1 hour at room temperature. Anti-β-actin antibody (1:5000 dilution; cat no. ab8226, Abcam), anti-SPAG5 antibody (1:2000 dilution, cat no. 14726-1-AP, Proteintech), anti-NOTCH1 (1:2000 dilution, cat no. ab52627, Abcam), anti-FOXM1 antibody (1:1000 dilution, cat no. ab207298, Abcam), anti-ADAM17 antibody (1:2000 dilution, cat no. sc-390859, Santa Cruz, Dallas, Texas, USA) and anti-HES1 (1:1000 dilution, cat no. ab108937, Abcam) antibody were used in this study. Then, the membranes were probed with the HRP-conjugated secondary antibodies at room temperature for 1 hour. Protein signaling was measured by using the ProfiBlot-48 (Tecan, Switzerland) with the help of ECL reagent (Millipore, USA) and quantified by using ImageJ software.

### CCK-8 assay

Cell proliferation was tested using the Cell Counting Kit-8 (CCK-8, Beyotime, Beijing, China) [28]. Cells were seeded in 96-well plates with 4, 000 cells in each well. After 24, 48 or 72 hours of cell transfection, cells were incubated with CCK-8 solution for 4 hours at 37°C. OD values at 450 nm were measured by a SpectraMax M2e (Molecular Devices, San Jose, CA, USA).

### Clone formation assay

The clone formation ability of cells was assessed using clone formation assay based on previously study [29]. The stably transfected cells were seeded in 6-well plates at 500 cells per well and incubated at 37°C for 14 days. The number of cell clones was counted under a microscope following being stained with crystal violet.

### EdU staining

Cell proliferation was also assessed by EdU staining [30]. In brief, MM cells (5 × 10^3^) were placed into each well of the 96-well plates. After 48 hours of transfection, the cells were incubated with 100 μL of EdU medium for 2 hours and fixed with 4% paraformaldehyde for 30 minutes. Then, the cells were penetrated by 0.5% Triton X-100 for 5 minutes and incubated with glycine for 5 min at room temperature and immersed in apollo dye reaction liquid for 30 minutes in the dark. Hoechst 33342 reaction was used for nuclear staining.

### Cell cycle assessment

Cells were fixed in ice-cold ethanol PBS for 4 hours at 4°C and re-suspended in ice-cold PBS containing 10 μg/mL propidium iodide and 10 μg/mL of DNase-free RNase (Sigma-Aldrich, MA, USA). Following incubation at 37°C for 30 min, cell cycle distribution was evaluated by flow cytometry analysis of nuclear DNA content with a flow cytometer (BD Biosciences) and Flowjo software [31].

### Cell migration and invasion detection

Cell migration and invasion were examined using the Transwell Chamber Assay [32]. Twenty-four hours after transfection, cells were plated in the upper chamber of the transwell (8.0-μm pore membranes, Corning, USA) in the serum-free medium at the density of 10^5^ cells/well. Cell culture medium with 10% FBS was added into the bottom chamber and served as a chemoattractant. After incubation at 37°C for 24 hours, the non-migrating cells were removed using swabs and the migrated cells were fixed with 4% formaldehyde and stained with crystal violet. Cell invasion assay was carried out using chambers pre-coated with 50 μL Matrigel (BD Bioscience) as described above. Following 48 hours of incubation, the cells were submitted to crystal violet staining. Cell numbers in five randomly selected fields were counted under a microscope.

### Immunofluorescence analysis

Immunofluorescence analysis was carried out based on previously described [33]. A total of 1× 10^4^ cells were plated on coverslips in 48-well plates and infected with shNC or shSPAG5. The cells were then incubated at 37°C for 24 hours and collected, and fixed with 4% paraformaldehyde for 30 min. Then, the cells were incubated with anti-E-cadherin antibody (cat no. ab11512, Abcam) at 4°C overnight. For Snail staining, the cells were penetrated with Trion-X 100 (Sigma-Aldrich, USA) and then incubated with anti-Snail antibody (cat no. ab167609). After that, the cells were probed with DAPI solution for 5 min. The cells were photographed using an immunofluorescence microscope (Olympus, USA).

### Animal experiments

The animal studies were performed according to previously reported [34] and approved by the Animal Care Committee of Shenzhen People’s Hospital. A375 cells (1 × 10^6^) stable transfected with shNC or shSPAG5 were injected into the armpit of 6-week male BALB/c nude mice (5 mice in each group). Four weeks later, mice were euthanized and weighted. Tumor volume was calculated by using the formula, V = Length×Width^2^/2. Then, the tumors were collected for IHC staining according to the above described. In addition, the lung tissues of mice were collected for hematoxylin eosin staining to assess the lung metastatic tumors [35,36].

### Statistical analysis

Three independent experiments were carried out in the current study. Statistical analysis was performed by using GraphPad Prsim 6. Student’s t-test and one-way ANOVA followed by Tukey’s tests were used for data analysis between two and multiple groups, respectively. A *p* < 0.05 was thought as statistical significance.

## Results

### SPAG5 expression was increased in MM tissues and cells

**Figure 1.**
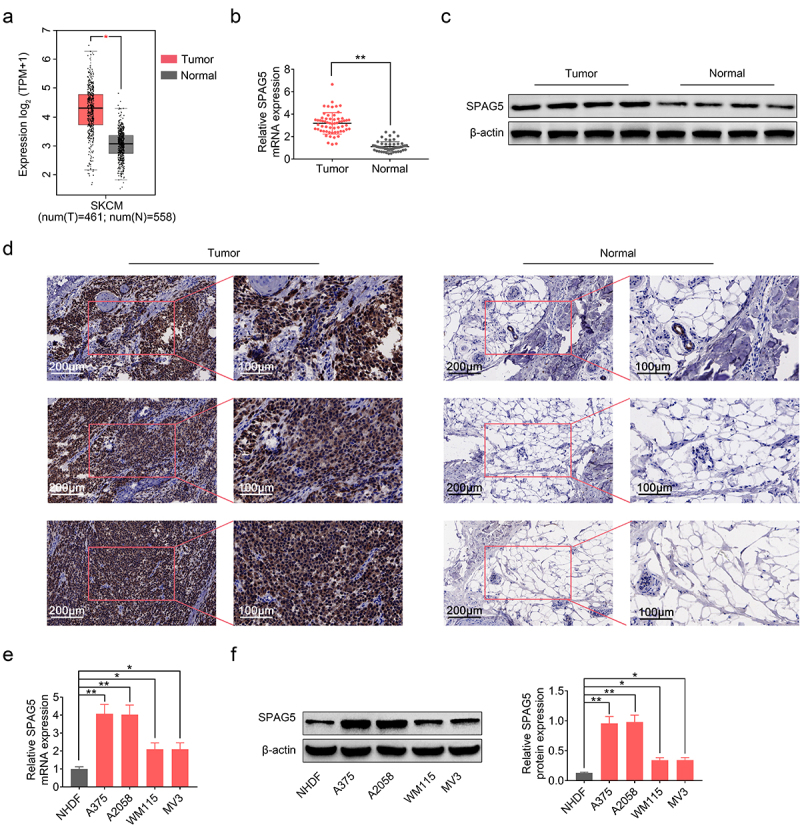
SPAG5 expression was increased in MM tissues and samples. (a) SPAG5 expression in MM tissues and normal tissues, as predicted in GEPIA database. (b) SPAG5 mRNA levels in 60 paired MM tissues and normal tissues were determined using qRT-PCR. (c, d) The protein levels of SPAG5 in 3 paired MM tissues and normal tissues were determined using Western blotting and IHC analysis. (e, f) The mRNA and protein expression of SPAG5 in NHDF, A375, A2058, WM115 and MV3 cell were measured by qRT-PCR and Western blotting. (n = 3, *P < 0.05, **P < 0.01).

To explore the role of SPAG5 and the underlying mechanisms in MM, we first assessed its expression patterns in MM tissues and the adjacent normal tissues. The GEPIA (Gene Expression Profiling Interactive Analysis) database (http://gepia.cancer-pku.cn/) showed a higher expression level of SPAG5 in MM tissues compared with normal tissues (Figure 1(a)), which was then verified by qRT-PCR (2-fold change; Figure 1(b)), Western blotting (2.5-fold change; Figure 1(c)) and IHC staining (3-fold change; Figure 1(d)) in MM clinical tissues. Also, we assessed the expression of SPAG5 in MM cells. Compared with NHDF, SPAG5 expressions in MM cell lines, A375, A2058, WM115 and MV3 were increased about 1 ~ 3-fold at mRNA and protein levels (Figures 1(e,f)). These results showed a higher expression level of SPAG5 in MM tissues and cells compared with the normal tissues and cells.

### Silencing of SPAG5 repressed cell viability and induced a G1 phase arrest in MM

**Figure 2.**
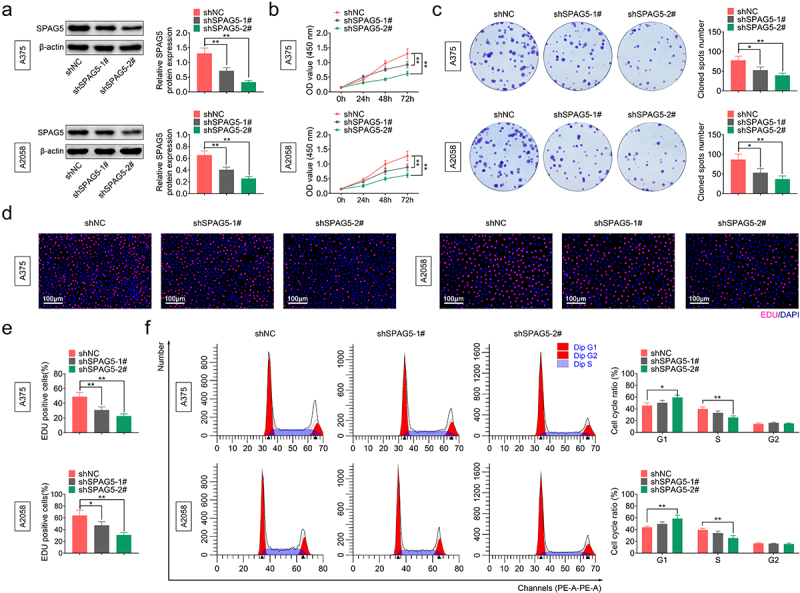
Deletion of SPAG5 inhibited MM cell growth and induced a G1 phase arrest. A375 and A2058 cells were transfected with shNC, shSPAG5-1 and shSPAG5-2, and submitted to the following assays. (a) SPAG5 expression was detected by using Western blotting assay. (b) CCK-8 was used for cell proliferation detection. (c) Colony formation assay was performed for cell viability test. (d) EdU staining was used for cell proliferation test. (e) Cell cycle distribution was assessed using flow cytometry assay. (n = 3, *P < 0.05, **P < 0.01).

To assess the role of SPAG5 in MM progression, we assessed the effect of SPAG5 downregulation on cell growth through the *in vitro* assays. Since A375 and A2058 cells had higher expression levels of SPAG5 than WM115 and MV3 cells, A375 and A2058 cells were used for the loss-of-function assays. Both shSPAG5-1# and shSPAG5-2# induced a 50–80% decrease in the expression level of SPAG5 (Figure 2(a)), and thereafter repressed cell growth (Figure 2(b)), colony formation (Figure 2(c)), and decreased the population of EdU-positive cells (Figure 2(d)) compared with the shNC group. In addition, shSPAG5-2# triggered G1 phase accumulation (from 42% to 60%) and decreased cell population of S phase (from 40% to 20%) in both A375 and A2058 cells (Figure 2(e)). These results demonstrated that silencing of SPAG5 repressed MM cell viability and accelerated G1 phase arrest, indicating that SPAG5 might serve as an oncogene in MM development.

### Silencing of SPAG5 inhibited MM cell migration and EMT

**Figure 3.**
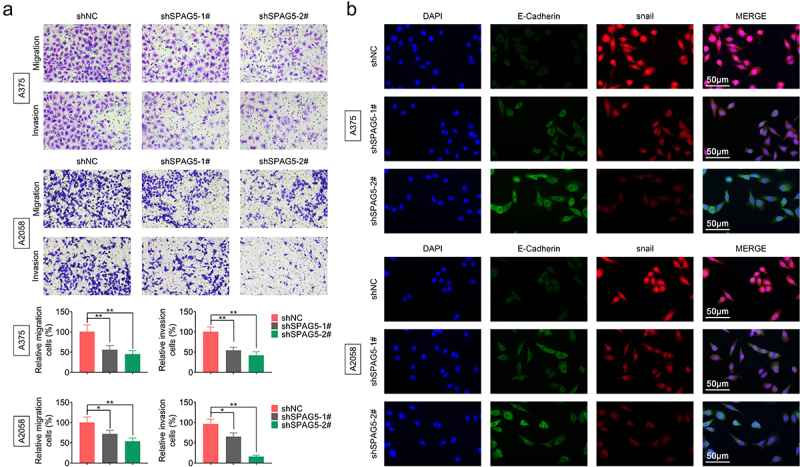
Deletion of SPAG5 expression repressed cell migration and EMT in MM. A375 and A2058 cells were transfected with shNC, shSPAG5-1 and shSPAG5-2, and submitted to (a) transwell assay to detect cell migration and invasion, and (b) immunofluorescence staining of E-cadherin and Snail. (n = 3, *P < 0.05, **P < 0.01).

Next, we assessed the effects of SPAG5 on regulating MM cell migration and EMT using the loss-of-function assays to further reveal SPAG5 role in MM progression. Compared with the shNC group, cell migration and invasion abilities were significantly impaired by about in half in A375 and A2058 cells (Figure 3(a)). Also, downregulation of SPAG5 decreased Snail expression to approximately 50%, while induced a 2-fold increase in E-cadherin expression compared with the shNC group (Figure 3(b)). These results heighted that downregulation of SPAG5 inhibited MM cell migration and EMT, further suggesting that SPAG5 serves as an oncogene in MM development.

### SPAG5 inactivated ADAM17/NOTCH signaling through FOXM1 in MM cells

**Figure 4.**
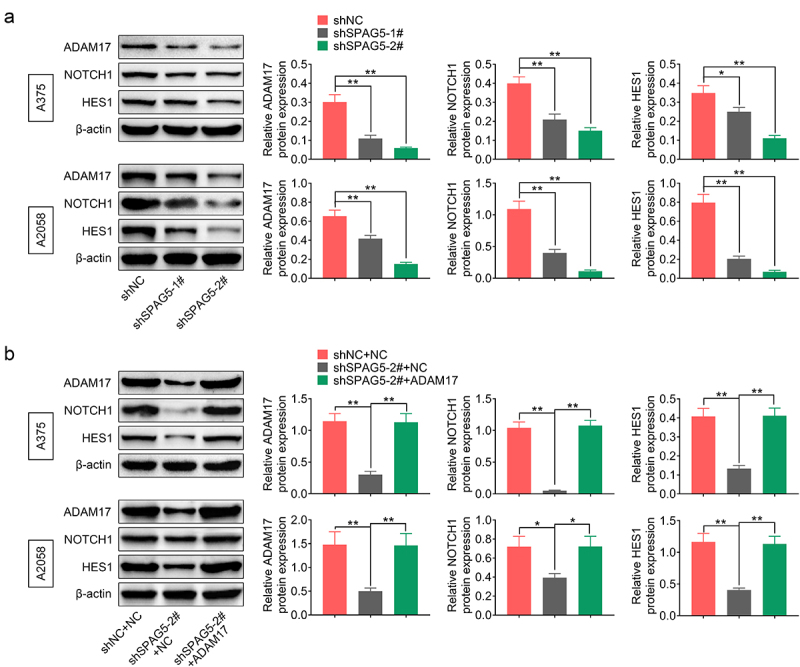
Downregulation of SPAG5 declined the expression of ADAM17, NOTCH1 and HES1 in MM cells. (a) Western blotting was used to detect the expression of ADAM17, NOTCH1 and HES1 in A375 and A2058 cells transfected with shNC, shSPAG5-1 and shSPAG5-2. (b) Western blotting was used to detect the expression of ADAM17, NOTCH1 and HES1 in A375 and A2058 cells transfected with shNC+NC, shSPAG5-2+ NC and shSPAG5-2+ ADAM17. (n = 3, *P < 0.05, **P < 0.01; NC, negative control).

**Figure 5.**
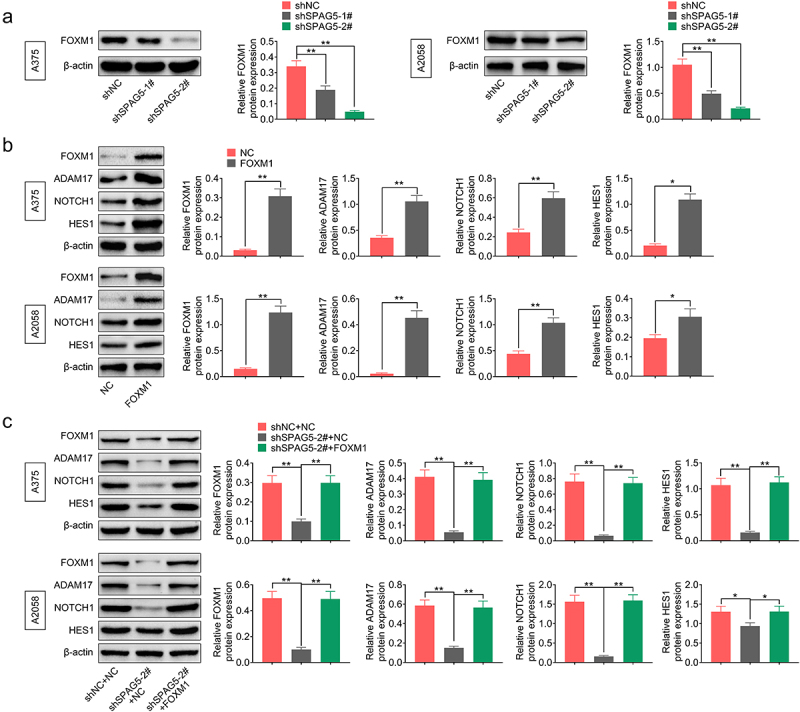
Downregulation of SPAG5 decreased the expression of ADAM17, HES1 and NOTCH1 through downregulation of FOXM1 in MM. (a) SPAG5 downregulation on the expression of FOXM1 was determined by Western blotting. (b)The expression of FOXM1, ADAM17, HES1 and NOTCH1 were detected by Western blotting in A375 and A2058 cells with FOXM1 overexpression or without. (c) Western blotting was used to detect the expression of FOXM1, ADAM17, NOTCH1 and HES1 in A375 and A2058 cells transfected with shNC+NC, shSPAG5-2+ NC and shSPAG5-2+ FOXM1. (n = 3, *P < 0.05, **P < 0.01; NC, negative control).

To reveal the mechanisms underlying SPAG5 downregulation-mediated inhibition of MM progression, we then assessed whether SPAG5 could activate ADAM17/NOTCH signaling through FOXM1. The results showed that the expressions of ADAM17, NOTCH1 and HES1 were decreased to 20%~50% following SPAG5 downregulation in A375 and A2058 cells compared with the shNC group (Figure 4(a)), while this effect was attenuated by ADAM17 overexpression (Figure 4(b)). In addition, downregulation of SPAG5 induced a 50 ~ 80% decrease in FOXM1 expression (Figure 5(a)). Overexpression of FOXM1 increased the expressions of ADAM17, NOTCH1 and HES1 (Figure 5(b)), and impaired the effects of shSPAG5 on downregulating of ADAM17, NOTCH1 and HES1 expression (Figure 5(c)). These findings suggested that downregulation of SPAG5 decreased FOXM1, ADAM17 and NOTCH1 expression in MM cells.

### Downregulation of SPAG5 expression decreased the tumorigenesis of MM cells

**Figure 6.**
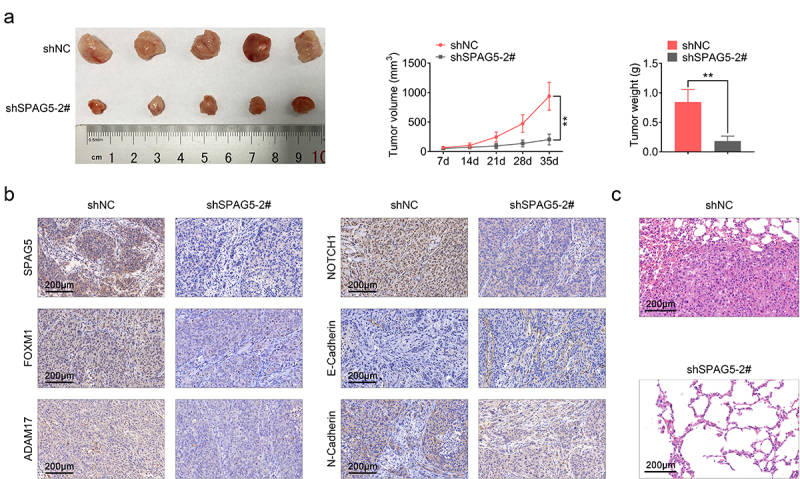
Downregulation of SPAG5 weakened the *in vivo* tumor formation ability of A375 cells. (a) Tumor volume and weight. (b) The expression of SPAG5, FOXM1, ADAM17, NOTCH1, E-cadherin and N-cadherin in shNC and shSPAG5-2 groups were detected using IHC. (c) Hematoxylin eosin staining was used to assess the lung metastasis of MM (Each group had 5 mice, **P < 0.01).

Furthermore, we assessed the role of SPAG5 in MM progression *in vivo*. Compared with the shNC group, tumor volume and weight were decreased to about 20% following SPAG5 downregulation (Figure 6(a)). Meanwhile, the expression levels of SPAG5, FOXM1, ADAM17, NOTCH1 and N-cadherin were decreased, and E-cadherin expression was increased (Figure 6(b)). In addition, downregulation of SPAG5 inhibited the lung metastasis of MM (Figure 6(c)). These results demonstrated that SPAG5 downregulation repressed tumor growth and metastasis.

## Discussion

SPAG5, encoding a mitotic spindle-related protein, has been reported to be implicated in carcinogenesis [37]. SPAG5 is consistently expressed at higher levels in many types of cancers, such as breast cancer [10,38,39], gastric cancer [13], cervical cancer [40], prostate cancer [12], ovarian cancer [41], osteosarcoma [18], hepatocellular carcinoma [42], bladder cancer [43] and lung cancer [44]. In this study, we first explored the role of SPAG5 in MM, and revealed that the expression of SPAG5 was upregulated in MM tissues and cells, providing a hypothesis that SPAG5 may be involved in MM progression.

From the loss-of-function assay, we found that silencing of SPAG5 inhibited cell growth, migration, and invasion abilities, induced G1 phase arrest and accelerated EMT in A375 and A2058 cells, as well as weakened the tumorigenesis of A375 cells *in vivo*. These results demonstrated that SPAG5 plays an oncogenic role in MM. Consistently, SPAG5 was reported to promote cancer progression in a variety of cancers, and targeting of SPAG5 might be a potent strategy for cancer treatment [42,43,45]. For example, SPAG5 promoted cell proliferation and reduced cell apoptosis in bladder urothelial carcinoma via regulating Wnt3/AKT/mTOR pathway [43]. Fe-doped chrysotile nanotubes carrying siSPAG5 leaded to significant repressions in the growth, migration, and invasion abilities of bladder cancer cells [45]. SPAG5 promoted the progression of hepatocellular carcinoma through CEP55-mediated activation of the PI3K/AKT pathway [42].

FOXM1, a transcription factor, has been demonstrated to express at higher levels in metastatic melanoma compared with primary melanoma, and the high expression of FOXM1 is associated with lower overall survival [16]. Impairment of FOXM1 activity weakened cell survival of MM [15]. Li et al. [18] demonstrated that SPAG5 could increase FOXM1 expression in osteosarcoma. We conjectured that SPAG5 may also modulate FOXM1 expression in melanoma. As respected, we observed that downregulation of SAPG5 decreased the expression level of FOXM1 in A375 and A2058 cells. In addition, FOXM1 can promote the expression of ADAM17, which targets the NOTCH1 signaling [20,46–48]. ADAM17 is a member of ADAMs family, which mediates the shedding and maturity of various membrane proteins such as HB-EGF, TNFα, NOTCH, and cadherins and is implicated in cancer progression [49]. In particular, ADAM17 serves as an executor of the cleavage of E-cadherin, leading to EMT and cell movement [50]. Through inhibiting the expression of ADAM17, rosmarinic acid repressed cell proliferation and migration, and enhanced cisplatin sensitivity in melanoma cells, highlighting a vital role of ADAM17 in melanoma progression [25]. Previous evidence has demonstrated that ADAM17/NOTCH1 is located at the downstream of FOXM1, and is modulated by SPAG5 [18,20,46–48]. In this study, we first explored the relationship between SPAG5 and FOXM1/ADAM17/NOTCH1 in MM. Consistently, our results demonstrated that downregulation of SPAG5 decreased the expression of FOXM1, ADAM17 and NOTCH1, which was counteracted by overexpression of FOXM1, suggesting that silencing of SPAG5 inhibited ADAM17/NOTCH1 signaling through downregulating of FOXM1 expression in MM cells. ADAM17/NOTCH1 signaling might be involved in SPAG5-mediated MM growth and migration. Nevertheless, we conjecture that NOTCH1 is not implicated in SPAG5/FOXM1/ADAM17-mediaited EMT, since ADAM17 acts as an executor of the cleavage of E-cadherin [50].

Moreover, we explored the role of SPAG5 in MM *in vivo*. Tumor volume and weight as well as the lung metastatic tumor number were significantly decreased when SPAG5 was downregulated in A375 cells, further validating our notion that SPAG5 served as an oncogene in MM. In addition, we found that the expressions of FOXM1, ADAM17, NOTCH1 and N-cadherin were decreased in SPAG5-downregulated tumor tissues, while E-cadherin expression was increased, highlighting the role of SPAG5 in downregulation of the expression of FOXM1, ADAM17 and NOTCH1, and inhibiting EMT.

Several limitations in this study should be clarified. One is that we didn’t explore the clinical value of SPAG5 in MM, such as the relationship between SPAG5 expression and clinical characteristics and prognosis of patients. Another limitation is that we did not explore whether FOXM1-mediated ADAM17/NOTCH1 signaling is involved in SPAG5-mediated MM progression.

## Conclusion

Collectively, this study reveals that SPAG5 is overexpressed in MM. Downregulation of SPAG5 represses MM cell growth, migration and EMT, which may be induced by the inactivation of the FOXM1/ADAM17/NOTCH1 signaling. Our study enriches the role of SPAG5 gene in MM progression, which might be explored as a therapeutic target for MM. Future systemic biology analyses would shed more light on the underlying interactions of SPAG5 and FOXM1/ADAM17/NOTCH1 signaling, and could help in developing novel diagnostic and therapeutic strategies for MM management.

## Data Availability

All data generated or analyzed during this study are included in this published article.
